# Cancer Pain Management: Comprehensive Assessment and Nonopioid Analgesics, Part 1

**Published:** 2017-07-01

**Authors:** Rita J. Wickham

**Affiliations:** Rush University College of Nursing (Adjunct Faculty), Chicago, Illinois

## Abstract

Pain is still undertreated and thus a significant problem for at least half of all cancer patients. Inadequately managed cancer pain may cause significant morbidity and even affect mortality, as well as patient quality of life. One enduring problem is suboptimal pain education in basic and advanced educational programs, and many myths and knowledge gaps persist. This article focuses on identifying and dispelling myths, thorough baseline and ongoing pain assessment, pain documentation, and interprofessional collaboration. It includes a comprehensive review of appropriate use of nonopioid analgesics—nonsteroidal anti-inflammatory agents and acetaminophen, and so-called adjuvant analgesics, such as antidepressants, anticonvulsants, and other drugs.

About 50% of cancer patients have undertreated pain, and the percentage is even greater among minority patients (Fisch et al., 2012; Paice & Von Roenn, 2014; Stein, Alcaraz, Kamson, Fallon, & Smith, 2015). Unrelieved pain is not inconsequential and can alter immunity and organ function, increase anxiety and depression, affect well-being and quality of life, and may even hasten death (Glare et al., 2014; Paice, 2010). Educational gaps and other barriers continue to affect interprofessional and oncology advanced practitioners’ (APs) roles in pain management for patients undergoing active cancer treatment or receiving palliative or survival care. This article is the first part of a two-part series centering on analgesics and will discuss barriers to cancer pain management, comprehensive assessment, and nonopioid analgesics. The focus of the second part, which will appear in a future issue of *JADPRO*, will be opioid analgesics. Nondrug measures are essential to optimal cancer pain control but are beyond the scope of this series. 

There are persistent gaps in pain management content in prelicensure and postgraduate health-care education (Institute of Medicine, 2011; Duke, Haas, Yarbrough, & Northam, 2013; Hunter et al., 2008; Murinson et al., 2013). Furthermore, professional and patient barriers still impede optimal cancer pain control (Breuer, Fleishman, Cruciani, & Portenoy, 2011; Kwon, 2014; Paice & Ferrell, 2011; Stein et al., 2015; Vallerand, Collins-Bohler, Templin, & Hasenau, 2007). Possible clinician barriers include inadequate knowledge regarding pain management principles, incorrectly held beliefs about adverse effects and addiction, little access to and collaboration with supportive care services, and concerns about legal and regulatory restrictions. Among patients, minority and elderly individuals are *more* likely to be uncomfortable talking to providers about their pain, to believe pain signals worsening cancer, to believe pain cannot be relieved, to have unaddressed concerns about addiction and side effects, and to be unable to pay for analgesics.

Sociopolitical factors are a recurring barrier, as spotlighted by Von Gunten (2016): "...A common belief among the public, including physicians, is that an opioid like morphine, even if prescribed by a physician for a medical indication, causes addiction" (p 348). The "evidence" for this myth is oft quoted and supported by flawed statistics that mistakenly calculate the rate of addiction as: 

 *Number of opioid addicts first introduced to opioids as prescription drugs *

 *All opioid addicts* 

This quotient is an erroneously estimated addiction rate of 60% to 100%. The correct calculation is:

 *All people with pain treated with an opioid who become addicted* 

 *All people with pain and treated with an opioid* 

This quotient reflects the actual iatrogenic risk for addiction or substance abuse of legitimately and appropriately prescribed opioids as 0.01% to 4%. 

According to the Substance Abuse and Mental Health Services Administration (SAMHSA), 75% of prescription opioid abusers are taking a family member’s or friend’s opioids or buying them on the street (Center for Behavioral Health Statistics and Quality, 2015; Cicero, Ellis, Surratt, & Kurtz, 2014). This is particularly salient given the highly publicized recommendations from the Centers for Disease Control (CDC) to limit opioid prescriptions for chronic pain (Dowell, Haegerich, & Chou, 2016). The CDC report explicitly excludes cancer pain, but the surrounding press may reinforce negative (and emotional) misconceptions about using opioid analgesics, even among cancer patients or their families. For instance, the CDC recommendations were followed by the Comprehensive Addiction and Recovery Act (CARA), which has implications for APs and patients (Viale, 2016). In response, the American Society of Clinical Oncology (ASCO) published a policy statement supporting access to opioids for cancer pain (ASCO, 2016).

## BASELINE PAIN ASSESSMENT

Oncology clinicians know regular pain assessment and documentation are essential but may not incorporate them into routine practice (Paice & Ferrell, 2011; Wells, McDowell, Hendricks, Deitrich, & Murphy, 2011). Many patients report that clinicians do not even ask about pain. Comprehensive assessment is the foundation for a pain management plan (drug and nondrug), reevaluation, and subsequent modifications in the plan.

**Location**

As many as 80% of cancer patients have multiple sites of pain; therefore, APs should ask patients to identify all painful sites and determine whether each is localized or spreads (radicular or referred; Caracenia & Portenoy, 1999). It is easier to pinpoint somatic pain (e.g., pathologic fractures), but visceral pain may be vague or referred to overlying skin or a distant site. For example, aching or gnawing right shoulder pain may be referred from hepatomegaly; left back pain may arise from a pancreatic tumor; and diffuse abdominal pain may be caused by omental inflammation, bowel or duct obstruction, hollow viscera stretching, or ascites, ischemia, or hepatomegaly (Shaiova, 2006). About 13% of patients with spinal metastases have radicular pain along affected dermatomes of distorted or compressed nerve roots, and some have accompanying motor or sensory deficits and hyperreflexia (Chang, Janian, Jain, & Chau, 2006). Thus, a metastatic deposit in the right side of the lumbar one vertebra (L1) could cause waist-level back pain radiating to the right hip and groin. Percussion of involved vertebrae usually elicits pain or tenderness. 

**Quality**

Asking "What does your pain feel like?" elicits details about pain quality that may aid analgesic selection (Table 1). Patients often use more than one quality word, reflecting different pain mechanisms (nociceptive or neuropathic) and analgesic choices (Chang et al., 2006; Dworkin, Jensen, Gammaitoni, Olaleye, & Galer, 2007; Holtan & Kongsgaard, 2009; Mackey et al., 2012; Pasternak, 2014). Visceral pain might be accompanied by nausea and vomiting, anorexia, bloating, and diaphoresis (Matthie & McMillan, 2014; Shaiova, 2006). Patients with neuropathic pain may have paresthesia (numbness or pins and needles), allodynia (pain evoked by a nonpainful stimulus, such as clothing or sheets touching the skin), dysesthesia (unusual or strange sensations described as painful), or autonomic changes (mottled or pink skin) over the painful area (Gilron, Tu, & Holden, 2013; Kerba, Wu, Duan, Hagen, & Bennett, 2010). 

**Table 1:**
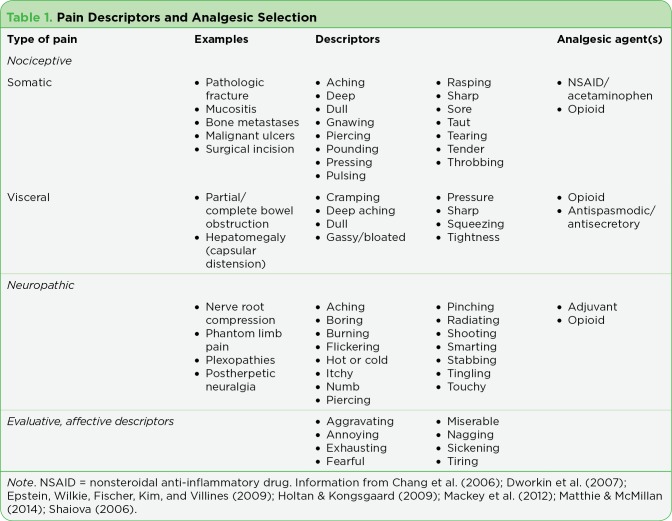
Pain Descriptors and Analgesic Selection

**Intensity**

Numerical rating scales (NRS) such as 0 to 10, and verbal rating scales (VRS) such as none, mild, moderate, or severe, are reliable, valid, and sensitive pain-intensity measures that most people understand easily (Brunelli et al., 2010; Hartrick, Kovan, & Shapiro, 2003; Hjermstad et al., 2011). The Faces Pain Scale–Revised (FPS-R) is similarly psychometrically sound and may be a useful alternative for some patients (Ferreira-Valente, Pais-Ribeiro, & Jensen, 2011; Swarm et al., 2010). An initial strategy may be asking a patient to rate pain severity on two scales to help them identify the scale they best understand. The Breakthrough Pain (BTP) Assessment is a 14-item list specific to BTP (Webber, Davies, & Cowie, 2016).

**Temporal Pattern of Pain**

Another assessment goal is to clarify temporal aspects: whether pain is constant or intermittent, what *aggravating* or *alleviating factors* increase or decrease pain, or whether pain is *paroxysmal (breakthrough)* or *end-of-dose failure*. Constant pain requires around-the-clock (ATC) analgesics. Up to 66% of cancer patients with well-controlled baseline pain have episodes of severe breakthrough pain—sudden, unpredictable, and spontaneous, or predictable incident pain provoked by specific triggers (e.g., weight bearing, coughing, or wound changes); these pains peak in 3 to 10 minutes and last about 30 minutes (Caraceni, Martini, Zecca, & Portenoy, 2004; Daeninck et al., 2016; Davies et al., 2013; Greco et al., 2011). End-of-dose failure occurs with sustained-release (SR) or transdermal opioids, when pain returns earlier than predicted with every dose. For example, a patient taking morphine SR every 12 hours experiences pain 10 hours after every dose (more common with generic SR products) is managed by changing morphine SR to every 8 hours.

**Effects of Pain on Mood, Activities, and Sleep**

The AP should ask the patient whether pain interferes with any daily activities, roles, work, enjoyment of life, emotional functioning, and sleep (McMillan, Tofthagen, & Morgan, 2008; Tavoli, Montazeri, Roshan, Tavoli, & Melyani, 2008; te Boveldt et al., 2013). These interrelated effects are often associated with depression and can occur even with mild pain but are magnified with moderate and severe pain. 

**Analgesic History**

An accurate analgesic history should identify analgesics discontinued because of unmanageable side effects or ineffective analgesia at maximized doses. Reviewing the patient’s current analgesics with them confirms doses taken (baseline and as needed [PRN]). For example, if another prescriber instructed the patient with pain to increase baseline or take more frequent PRN doses, the prescriber may not have documented this in the patient’s medical record (MR). Other patients do not take prescribed opioids because of unaddressed concerns or unmanaged adverse effects (Gunnarsdottir, Donovan, Serlin, Voge, & Ward, 2002; Kwon, 2014). 

**Patient’s Goal for Pain Relief**

The AP should ask and document the patient’s estimate of acceptable pain level/relief (Hui & Bruera, 2014). Most patients state mild pain is tolerable, and few request complete pain relief (Dalal et al., 2012). This is also the time to explore patient beliefs and concerns about pain and analgesia, which may be influenced by culture, religion, adverse effects, worries about addiction, paying for analgesics, or even opioid availability at local pharmacies (Kwon, 2014; Situ, Wang, Shao, & Zhu, 2012). 

At reassessment, asking the patient whether current pain control is "good enough" or "could be a little better" aids in tweaking analgesic doses. Another way to determine this is to ask whether their pain is worse, the same, or better than at the last assessment and to quantify this with a relief scale (0% to 100%, or none, slight, moderate, or complete) to evaluate analgesia (Gilron & Jensen, 2011). Any improvement helps patients to focus on positive aspects of a management plan and increases their confidence that further pain relief is possible. It may be helpful for the patient to reframe the situation by asking: "What can you do now that you could not do before starting this pain medicine?" Questions about any new and bothersome side effects—the other side of the efficacy coin—are equally important to analgesic planning. 

**Risk Assessment for Substance Use**

Exploring any personal or family history of alcohol or drug use or diagnosed major psychiatric disorder should be routine, as such factors may signify increased risks for aberrant drug-taking behaviors (Barclay, Owens, & Blackhall, 2014; Portenoy & Ahmed, 2014). Other clues might reflect risk, such as having a cancer associated with heavy alcohol use or smoking (e.g., head and neck cancer), being a current heavy smoker, having a history of automobile accidents or prolonged unemployment, or having a limited support system. Conversely, patients with limited prognoses or those who are in recovery programs are at lower risk. It is critical to ask patients about drug and alcohol use, rather than accepting comments about "drug-seeking behaviors" and situations and any treatment interventions. Most people will be truthful, but asking exaggerated questions such as, "Do you use recreational heroin?" or "Do you drink two bottles of wine or hard liquor or a case of beer each day?" usually prompts honest answers about less extreme use. 

There are no universally accepted definitions of substance abuse (use disorder) and addiction; however, some definitions for substance abuse and addiction, as well as dependence, tolerance, and drug diversion or pseudoaddiction, are summarized in Table 2. The current American Psychiatric Association (APA) diagnostic labels of "substance use disorder" and "addiction" are not interchangeable (APA, 2013; Hartney, 2016). The APA criteria for substance use (e.g., alcohol, cannabis, hallucinogens, inhalants, opioids, anxiolytics, and tobacco) disorders are categorized from mild to severe (Norko & Fitch, 2014). The American Pain Society, the American Society of Addiction Medicine, and the American Academy of Pain Medicine agree that addiction is a complex problem and is not synonymous with dependence and tolerance (American Society of Addiction Medicine, 2011; Heit, 2003; Volkow & McLellan, 2016). 

**Table 2:**
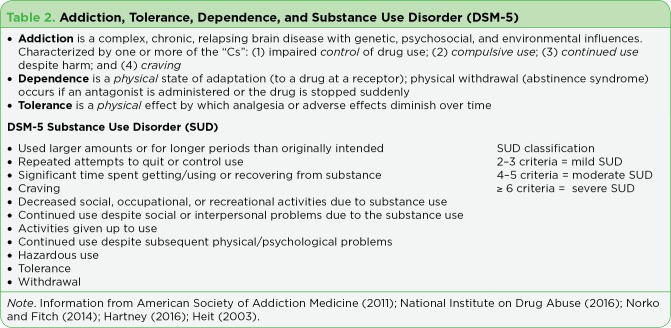
Addiction, Tolerance, Dependence, and Substance Use Disorder (DSM-5)

Weissman and Haddox (1989) coined the term "pseudoaddiction" to describe labeling patients as "drug-seekers" or "addicts" because they ask for larger-than-ordered or more frequently-than-prescribed opioid doses when prescriptions are inadequate. Clinicians often mistrust "pseudoaddicted" patients, leading to adversarial relationships. On the other hand, a clinician who strongly suspects a patient of diversion (a legal problem) *must* stop prescribing drugs with abuse potential and investigate the situation (Portenoy & Ahmed, 2014). 

Dependence and tolerance are physical phenomena but are *not* addiction. Continued opioid occupancy at receptors results in physical dependence, which occurs with steroids and some other drugs. This means withdrawal syndrome (e.g., anxiety, insomnia, agitation, abdominal cramping, etc.) will occur if an opioid antagonist (e.g., naloxone) is administered or an opioid is abruptly stopped rather than titrating doses downward. Tolerance is also physical, whereby adverse effects resolve (tolerance develops) or analgesia declines; this is a rare event in cancer patients, who almost always need larger doses because of increased, tumor-related pain (Ripamonti, Santini, Maranzano, Berti, & Roila, 2012). Unfortunately, the APA substance use criteria include tolerance and withdrawal, and other criteria (using larger amounts for longer periods, attempts to quit or control use) can be subjectively defined in some situations.

Advanced practitioners should explore patient concerns about becoming "hooked" to opioids, having unmanaged side effects, or believing that opioids are a "last resort" or hasten death (Bedard et al., 2013; Gunnarsdottir et al., 2002; Kwon, 2014; Reid, Gooberman-Hill, & Hank, 2008). There is evidence that *poorly controlled* pain actually shortens life, and taking opioids for cancer pain may *increase* survival (Halabi et al., 2008; Minami, Fujimoto, Ogata, Yamamoto, & Komuta, 2015; Portenoy et al., 2006). Family members may hold similar or more negative beliefs and influence a patient’s willingness or ability to take analgesics. Addressing family learning needs and dispelling concerns should thus be part of a pain management plan (Vallerand et al., 2007). 

## ANALGESICS

The analgesic ladder was intended to address inadequate cancer pain management in underdeveloped countries and guide step-wise analgesics for mild pain—a step 1 nonsteroidal anti-inflammatory drug (NSAID) or acetaminophen (APAP); a step 2 "weak" opioid for moderate pain; or a step 3 "strong" opioid for severe pain—with an adjuvant analgesic, as indicated, at any step (World Health Organization, 1987). Suggested changes include adding new analgesics, a two-step approach with small morphine-like opioid doses for moderate pain, or adding a pain intervention step (Eisenberg, Marinangeli, Birkhahm, Paladín, & Varrassi, 2005; Maltoni et al., 2005; Vargas-Schaffer, 2010). The ladder approach is helpful to frame analgesic "educated guesses" but is not exact as 24% to 30% of patients do not attain "best" pain relief (Stute, Lehmann, & Grond, 2001; Zech, Grond, Lynch, Herte, & Lehmann, 1995). 

## NONOPIOID ANALGESICS: NSAIDs AND ACETAMINOPHEN

Over-the-counter (OTC) and prescription NSAIDs are useful for mild to moderate pain with an inflammatory component (e.g., bone metastases or fungating lesions). Regularly scheduled NSAIDs should be taken for maximal efficacy, and adding an NSAID to an opioid for severe pain may enhance analgesia and allow lower opioid doses. The NSAIDs interrupt cyclooxygenase (COX) enzyme (COX-1 and COX-2) conversion of arachidonic acid to prostaglandins and thromboxanes, which may modulate, intensify, or maintain pain (Paice & Ferrell, 2011; Pountos, Georgouli, Bird, & Giannoudis, 2011). In addition, COX-1 and COX-2 have important roles in normal organ function and homeostasis (Kirkby et al., 2013). 

All NSAIDs except celecoxib are nonselective (Table 3) and bind with varying potencies to COX-1 and COX-2 (Grosser, 2009; James & Cleland, 2006). Aspirin (ASA), the prototypic nonselective NSAID, irreversibly inhibits COX activity for its duration in particular target tissues (Knights, Mangoni, & Miners, 2010; Munir, Enany, & Zhang, 2007). For instance, repeated aspirin doses ≥ 30 mg/day cause cumulative and dose-related platelet inhibition, with recovery 8 to 12 days after aspirin is stopped. Platelet effects from other NSAIDs are reversible, and times to recovery differ. 

**Table 3:**
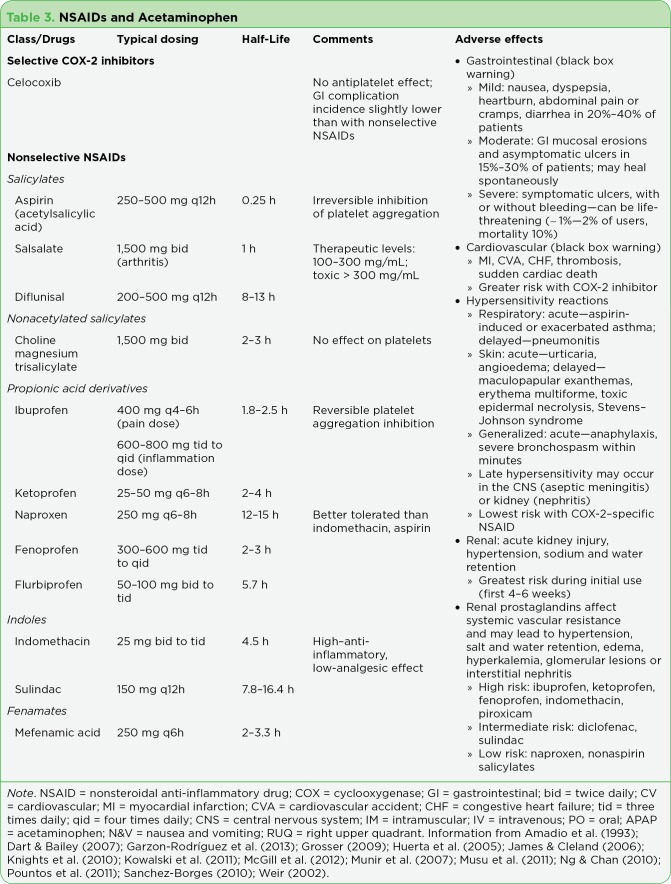
NSAIDs and Acetaminophen

**Table 3a:**
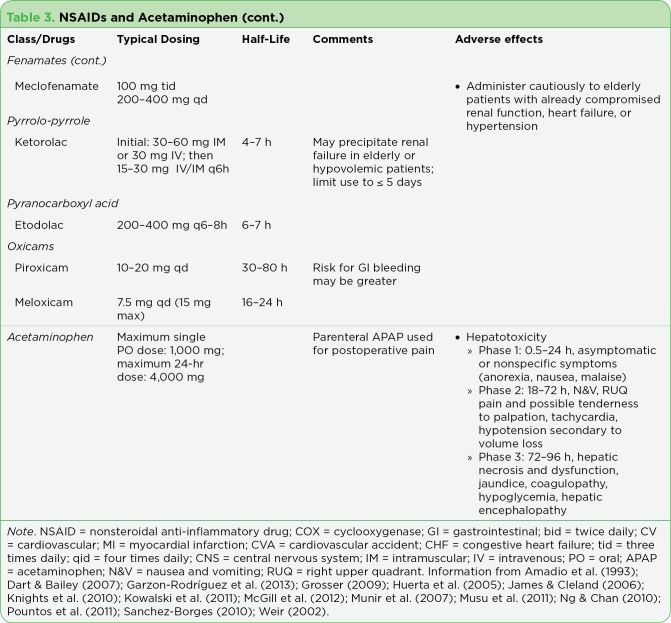
NSAIDs and Acetaminophen (cont.)

The NSAIDs (except ketorolac) are relatively inexpensive. The NSAID half-lives vary; a longer half-life means less frequent dosing but may increase the risk for adverse effects, especially in elderly or unhealthy people (Amadio, Cummings, & Amadio, 1993). The NSAIDs have an analgesic ceiling, above which only toxicity increases. A safe strategy is topical NSAID application over a painful site, which may decrease pain with little risk for systemic absorption and adverse effects (Pountos et al., 2011).

**Adverse Effects of NSAIDs**

Major NSAID adverse effects are gastrointestinal (GI), cardiovascular (CV), hypersensitivity, and renal events. The NSAIDs can damage gastric, small bowel, and colonic mucosa (Ng & Chan, 2010). Risk factors for GI events are age > 65, tobacco or alcohol use, history of peptic ulcer, longer NSAID use, use of two or more NSAIDs, and poor performance status (Pountos et al., 2011). A proton pump inhibitor (PPI; e.g., omeprazole) or histamine-2 (H2) blocker (e.g., famotidine) decreases ulcer incidence but does not prevent severe GI complications. 

Cardiovascular complications (e.g., myocardial infarction, stroke, systemic and pulmonary hypertension, congestive heart failure, and sudden cardiac death) are more likely with COX-2 selective (celecoxib) than nonselective NSAIDs (Grosser, 2009). Mechanisms are unclear but may relate to venous thromboembolic, blood pressure, or other effects (García Rodríguez, Tacconelli, & Patrignani, 2008). Acetaminophen or an opioid may be a safer analgesic for patients with significant CV disease (Vardeny & Solomon, 2008).

The NSAIDs cause 21% to 25% of all drug-induced respiratory, skin, or generalized hypersensitivity reactions (Kowalski et al., 2011). Acute hypersensitivity arises immediately to several hours after ingestion, and delayed hypersensitivity occurs > 24 hours later. Patients with hypersensitivity to one NSAID should never take moderate to strong COX inhibitors but generally tolerate weak inhibitors such as acetaminophen, celecoxib, salsalate, and trisalicylate (Kowalski et al., 2011; Sanchez-Borges, 2010).

Renal toxicity occurs in < 1% of relatively healthy people taking a selective or nonselective NSAID but accounts for 7% of all cases of reversible or nonreversible acute renal failure (Musu et al., 2011; Pountos et al., 2011). Renal damage is most common in the first 4 to 6 weeks, but the risk continues with long-term use. The elderly are at greatest risk because of decreased glomerular filtration rate, comorbid conditions (CV disease, hypertension, or diabetes), and taking associated drugs (e.g., β-blockers, angiotensin-converting enzyme (ACE) inhibitors, or diuretics; Huerta, Castellsague, Varas-Lorenzo, & Rodríguez, 2005; Weir, 2002).

**Acetaminophen**

Acetaminophen weakly inhibits COX-2 in the central nervous system, and is about as effective for pain and fever as aspirin, but has no anti-inflammatory action (Twycross, Pace, Mihalyo, & Wilcock, 2013). Acetaminophen is well tolerated if daily doses do not exceed 4,000 mg, and single doses are ≤ 1000 mg (Munir et al., 2007). Dart and Bailey (2007) reviewed 791 research articles that included 40,202 patients taking APAP, 77% in prospective and 23% in retrospective studies. No patient in any prospective study who took APAP at 3.9 to 4 g/day for ≥ 24 hours suffered acute liver failure (ALF), underwent liver transplantation, or died. On the other hand, 32 (0.3%) of those in retrospective studies had ALF, 1 (0.01%) required liver transplantation, and 6 (0.06%) died. Conclusions were that APAP ≤ 4 g/day is not hepatotoxic, and differences in retrospective studies were probably due to mistaken recall and reporting bias or inadvertent overdoses. In November 2015, the US Food and Drug Administration (FDA)’s final guidance set the maximum APAP daily dose as 4,000 mg (rather a specific number of pills), as well as 325 mg in any combination product. The FDA warned liver damage might occur with higher doses if APAP is taken with other APAP-containing drugs or by persons with an alcohol consumption of ≥ 3 drinks/day (US Food and Drug Administration, 2016). 

The minimum *single* hepatotoxic dose of APAP is 7.5 g, but repeated doses of combination APAP products (*supratherapeutic overdose*) cause about 50% of ALF cases (Larson et al., 2005; Temple & Baggish, 2005). Chronic alcoholism or liver disease increases the risk after acute overdose, but hepatotoxicity can occur in patients without liver disease. Overdoses overwhelm the minor APAP metabolic pathway, causing accumulation of n-acetyl-p-benzoquinone imine, a hepatotoxic intermediate metabolite (McGill et al., 2012). In rare instances, liver toxicity progresses to renal failure, multiorgan failure, and death. N–acetylcysteine administered within 8 hours after a toxic APAP dose is most effective but can be beneficial even ≥ 24 hours later (Farrell, 2016).

## ADJUVANT ANALGESICS

Adjuvant (coanalgesics) have other primary indications but have been shown to decrease pain with diabetic neuropathy, postherpetic neuralgia, or HIV-related neuropathy. A few studies have focused on cancer-related neuropathic pain (Jongen et al., 2013; van den Beuken-van Everdingen et al., 2016). Table 4 includes Canadian Pain Society first-line coanalgesics: tricyclic antidepressants (TCAs), serotonin norepinephrine-reuptake inhibitors (SNRIs), and gabapentinoids (Moulin et al., 2014). Lidocaine 5% patches or later-line adjuvants (e.g., older anticonvulsants, corticosteroids, and *N*-methyl-D-aspartate [NMDA] receptor antagonists) may help some patients (Vadalouca et al., 2012; Mitra & Jones, 2012). 

**Table 4:**
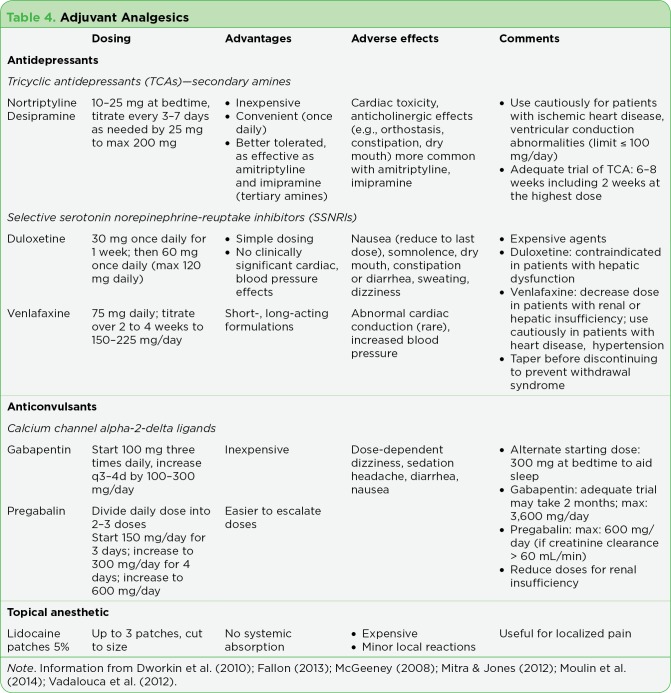
Adjuvant Analgesics

**Antidepressants**

A meta-analysis of many small TCA studies confirmed about one-third of patients experienced almost 50% relief of neuropathic pain, and only 4% had dose-limiting adverse effects (Vadalouca et al., 2012). The TCAs may also alleviate anxiety and insomnia (Fallon, 2013). The TCAs inhibit norepinephrine and serotonin reuptake at dorsal spinal cord synapses and secondarily block neural sodium channels and NMDA glutamate receptors (Mitra & Jones, 2012). The TCAs, including secondary (desipramine and nortriptyline) and tertiary amines (amitriptyline and imipramine), have similar analgesic efficacy. However, tertiary TCAs have worse anticholinergic effects (dry mouth, constipation, and orthostasis), sedation, and cardiac effects—particularly amitriptyline, which should be used cautiously in elderly patients with congestive heart failure, ischemic heart disease, cardiac arrhythmia, or bundle branch block.

Duloxetine and venlafaxine, selective SNRIs, are effective for neuropathic pain and have fewer adverse effects than TCAs (McGeeney, 2008; Vadalouca et al., 2012). Duloxetine dosing is simple: 60 mg once or twice a day is equally effective (Dworkin et al., 2010). Duloxetine does not cause clinically important electrocardiographic or blood pressure changes. Cardiovascular effects are rare with venlafaxine, which typically decreases pain after the dose is increased from 75 mg to ≥ 150 mg/day (Fallon, 2013). Venlafaxine may also reduce hot flushes and menopausal symptoms and may be particularly useful for women with breast cancer.

**Anticonvulsants**

Neuropathic pain is somewhat analogous to seizures; low levels of gamma-aminobutyric acid (GABA), changes in voltage-gated calcium or sodium channels, and downregulated spinal cord GABA receptors diminish inhibitory regulation of hyperexcitable, injured nerves that fire spontaneously (Mitra & Jones, 2012; Vadalouca et al., 2012). Gabapentin and pregabalin bind to and modulate voltage-gated calcium channels, inhibit neurotransmitter release, and stabilize neuronal cell membranes (Dworkin et al., 2010; Fallon, 2013; McGeeney, 2008). Either drug usually relieves postherpetic neuralgia, painful diabetic neuropathy, spinal cord injury pain, or neuropathic cancer pain relief within 1 to 2 weeks. 

Gabapentin is inexpensive and preferred by insurers, whereas pregabalin is more expensive and usually a second-line option for patients who do not tolerate gabapentin (Mitra & Jones, 2012). Gabapentin has poor oral bioavailability and nonlinear pharmacokinetics, so doses are escalated slowly (Dworkin et al., 2010; Vadalouca et al., 2012). The usual starting dose is 100 mg three times per day, but a single 300-mg dose at bedtime may aid sleep and minimize daytime sleepiness. Pregabalin has linear pharmacokinetics, so dosing is straightforward. Patients who tolerate the first dose level (150 mg/day) but do not attain pain relief can be increased to 300 mg/day after 1 week and to 600 mg/day a week later. 

**Topical Lidocaine**

Topical lidocaine 5% patches may be used alone or with other drugs and reduce ectopic voltage-gated sodium channel activity in damaged sensory nerves without affecting normal sensation (Fallon, 2013; Garzón-Rodríguez, Merchan, Calsina-Berna, Lopez-Romboli, & Porta-Sales, 2013; McGeeney, 2008). They are helpful for about 25% of patients with postherpetic neuralgia or other localized neuropathic pain, such as painful mastectomy or thoracotomy scar, or rib cage or subcutaneous tumor.

Up to three lidocaine patches are cut to shape and applied to *intact* skin for 12 hours, during which 5% of the patch dose is absorbed, and then removed for 12 hours (McGeeney, 2008). In practice, patches are often left on for longer times because patients fear return of pain if patches are removed, and adherence to the 12-hour on-and-off schedule may be difficult. Pharmacokinetic studies have confirmed four patches are safe and well tolerated for 3 consecutive days, whether reapplied every 12 or 24 hours (Gammaitoni, Alvarez, & Galer, 2002). No differences in plasma lidocaine concentrations with either application schedule were noted, and serum lidocaine levels reached only 14% of the antiarrhythmic dose. This lack of systemic effects means dose adjustments for renal or hepatic dysfunction are not necessary (Fallon, 2013; McGeeney, 2008). 

**Other Adjuvant Agents**

There are no randomized or adequately powered studies of second- or later-line adjuvant drugs (other anticonvulsants, corticosteroids, antispasmodics, cannabinoids, or ketamine), which might be tried if first-line adjuvant drugs are ineffective or intolerable, or for other indications (Dworkin et al, 2010). For instance, clonazepam, a long-acting benzodiazepine with anticonvulsant effects, is often helpful for myoclonic jerks as well as anxiety (Caviness, 2014; Van Zanducke, 2003). Slow escalation minimizes the risk for ataxia and sleepiness. Oxcarbazepine, carbamazepine, and topiramate are anticonvulsants that suppress sodium channel–mediated ectopic neuronal discharges, and baclofen, an antispasmodic, GABA-B agonist, is sometimes tried for neuropathic pain (McGeeney, 2008; Mitra & Jones, 2012; Vadalouca et al., 2012).

Corticosteroids are typically used for painful inflammation with serious conditions, such as tumor-related bony epidural compression or nerve root inflammation (Leppert & Buss, 2012; Mitra & Jones, 2012; Prommer, 2015; Vadalouca et al., 2012). Inflammation or compression can also be problematic with radiation therapy (RT)-induced "flare," particularly with whole-brain or spinal RT, from primary brain tumors or metastasis-induced cerebral edema, or hepatomegaly-related liver capsule pain.

Dexamethasone is most often used because of low mineralocorticoid and fluid retention effects as well as a long half-life (36–54 hours) allowing once-per-day dosing. Dexamethasone administration in the late afternoon or evening prevents corticosteroid stimulant effects and sleep disturbances (McGeeney, 2008). A small oral dose (dexamethasone 1 or 2 mg twice a day) may benefit patients with nonemergent problems such as pain poorly responsive to opioids and dose titration as needed. Conversely, patients with impending spinal cord compression or another emergent problem may be started on a higher dose (16 mg to 96 mg/day) and rapidly titrated down to a minimally effective dose (Prommer, 2015). Dose-related adverse effects may include hyperglycemia, hypertension, fluid retention, immunosuppression, GI, and neuropsychologic effects.

Hyoscine (scopolamine) or octreotide may alleviate colicky abdominal pain or spasms from partial or total bowel obstruction. Hyoscine has antispasmodic and local anticholinergic effects in the gut at smooth muscle muscarinic receptors; induces smooth muscle relaxation; and reduces pathologically enhanced peristalsis, gut motility, and cramping (Soares & Chan, 2007; Tytgat, 2007). Hyoscine does not cross the blood-brain barrier and has a low incidence of systemic anticholinergic adverse events. Continuous intravenous infusion, 60 mg over 24 hours, may control pain from inoperable bowel obstruction (Soares & Chan, 2007). Octreotide, a somatostatin analogue, reduces gastric, pancreatic and intestinal secretions, and GI motility. It is similarly effective as hyoscine for colicky pain and may have a more rapid onset.

Cannabis, which has been used medicinally for thousands of years, comprises numerous phytocannabinoids that may be synergistic with opioids and have antiemetic and appetite stimulating effects (Abrams, 2016; Fallon, 2013; Moulin et al., 2014). Exogenous and endocannabinoids bind to cannabinoid receptors (CB1 and CB2) throughout the central nervous system and periphery to influence intracellular signaling (Russo & Hohmann, 2015; Whiting et al., 2015).

Physiologic effects of cannabinoids include pain modulation at peripheral, spinal, and supraspinal levels, which decreases neuropathic pain, hyperalgesia, and inflammation. Cannabinoids are usually well tolerated, with gradual dose increases, but adverse effects may include dizziness, drowsiness, impaired psychomotor function, dry mouth, and dysphoria. Sativex, an oromucosal cannabinoid spray for cancer-related pain, is available in Canada and Europe. In the United States, cannabis is currently classified as a Schedule I substance (no accepted medical use and high abuse potential), which impedes research. The sociopolitical climate seems to be changing, and the number of states permitting medical (and recreational) marijuana use is increasing. Clinicians must know about cannabis status in their state and review research and clinical evidence for use, as well as differences in commercial products and marijuana (Savage et al., 2016). 

Ketamine, structurally similar to phencyclidine, is a dissociative anesthetic and noncompetitive NMDA antagonist in descending spinal inhibitory pathways (Niesters, Martini, & Dahan, 2013). Ketamine can induce dose-related psychotropic adverse effects (e.g., auditory or visual hallucinations, paranoid ideation, panic attacks, nightmares or vivid dreams, and an unpleasant or euphoric drug high). Cardiovascular effects (e.g., tachycardia and hypertension) can occur after low-dose ketamine infusion (Niesters et al., 2013). A subanesthetic dose trial may be done for difficult to control or intractable neuropathic, inflammatory, or ischemic pain resistant to other options (McGeeney, 2008). Loveday & Sindt (2015) reported that patients with intractable pain treated with subanesthetic, weight-based CIV ketamine (per university hospital protocol) usually had mild and transient side effects controlled with low-dose benzodiazepine or haloperidol. 

## CONCLUSION

Oncology APs, wherever they practice, have important roles and opportunities to optimize pain management in cancer patients. Collaborative, interprofessional roles with other APs, oncology pharmacists, oncologists, and others may identify personal or professional colleague knowledge gaps and potential learning opportunities, such as interprofessional pain management rounds.
